# Structure of a (3+1) hybrid G-quadruplex in the *PARP1* promoter

**DOI:** 10.1093/nar/gky1179

**Published:** 2018-12-15

**Authors:** Anjali Sengar, J Jeya Vandana, Vicki S Chambers, Marco Di Antonio, Fernaldo Richtia Winnerdy, Shankar Balasubramanian, Anh Tuân Phan

**Affiliations:** 1 School of Physical and Mathematical Sciences, Nanyang Technological University, Singapore 637371, Singapore; 2 Department of Chemistry, University of Cambridge, Cambridge CB2 1EW, UK; 3 Cancer Research UK Cambridge Institute, University of Cambridge, Li Ka Shing Centre, Cambridge CB2 0RE, UK

## Abstract

Poly (ADP-ribose) polymerase 1 (PARP1) has emerged as an attractive target for cancer therapy due to its key role in DNA repair processes. Inhibition of PARP1 in BRCA-mutated cancers has been observed to be clinically beneficial. Recent genome-mapping experiments have identified a non-canonical G-quadruplex-forming sequence containing bulges within the *PARP1* promoter. Structural features, like bulges, provide opportunities for selective chemical targeting of the non-canonical G-quadruplex structure within the *PARP1* promoter, which could serve as an alternative therapeutic approach for the regulation of PARP1 expression. Here we report the G-quadruplex structure formed by a 23-nucleotide G-rich sequence in the *PARP1* promoter. Our study revealed a three-layered intramolecular (3+1) hybrid G-quadruplex scaffold, in which three strands are oriented in one direction and the fourth in the opposite direction. This structure exhibits unique structural features such as an adenine bulge and a G·G·T base triple capping structure formed between the central edgewise loop, propeller loop and 5′ flanking terminal. Given the highly important role of PARP1 in DNA repair and cancer intervention, this structure presents an attractive opportunity to explore the therapeutic potential of PARP1 inhibition via G-quadruplex DNA targeting.

## INTRODUCTION

Poly (ADP-ribose) polymerase-1 (PARP1) is a nuclear enzyme known for its key role in the DNA repair processes (1). PARP1 binds to DNA breaks and recruits DNA repair proteins by catalyzing the formation of poly (ADP-ribose) scaffolds (1,2). Additionally, PARP1 has also been implicated in other cellular processes such as transcriptional regulation, chromatin remodelling, cell signaling and cell death (3–5). Synthetic lethality induced by PARP1 inhibition has emerged as a promising method for targeting tumor cells with defective homologous recombination pathways (6). Inhibition of PARP1 activity in homologous recombination deficient cancer cells causes accumulation of unrepaired single-strand DNA breaks which are potentially converted to double-stranded DNA breaks, causing cell death selectively in those cancer cells (7).

Consequently, several PARP1 inhibitors were developed showing promising results, with olaparib being the first one to be approved clinically for treating BRCA1/2 mutated cancers (8,9). However, the use of these drugs would be limited in light of increasing evidence showing acquired resistance to PARP1 inhibitors, mediated by multiple molecular mechanisms including changes in the expression of PARP1 itself (10). Alternative strategies are required to selectively regulate PARP1 expression that can be used in combination with current PARP1 inhibitors or alone.

A recent G-quadruplex-specific sequencing study (G4-seq) has identified non-canonical G-quadruplex-forming sequences in the promoter region of the *PARP1* gene (11), suggesting a potential for interfering with PARP1 regulation via G-quadruplex targeting. A link between ligand-mediated stabilization of G-quadruplexes in gene promoters and transcriptional regulation has been proposed for several oncogenes (12–14). Moreover, PARP1 has also been linked with transcriptional regulation of *c-KIT, c-MYC* and *KRAS* oncogenes by interacting with G-quadruplex structures in the promoter (15–17). Chemical inhibition of PARP1 synergized with the G-quadruplex ligand RHPS4 in HT29 xenografts could further inhibit tumor cell growth by preventing the repair of uncapped telomere ends (18). Transcriptional repression of PARP1 could, therefore, help in the prevention of diseases that occur due to overexpression of PARP1 and subsequently influence the expression of other oncogenes. To investigate the opportunity of G-quadruplex mediated PARP1 regulation, we set to confirm that the G-rich sequence element in the *PARP1* promoter indeed forms a stable G-quadruplex and if so, determine its folded structure. In particular, we aimed to elucidate unique structural features of the *PARP1* G-quadruplex that could, in principle, be exploited for selective chemical targeting of this promoter, given the therapeutic relevance of PARP1.

G-quadruplexes are generally four-stranded structures formed by G-rich nucleic acids comprising a stack of multiple guanine(G)-tetrads. A G-tetrad is a square planar arrangement of four guanines stabilized by Hoogsteen hydrogen bonds with a metal ion coordination in the center (19,20). A wide variety of G-quadruplex structures have been elucidated, differing in terms of strand orientations, glycosylic conformations, groove widths, connecting loops, molecularity and the number of tetrads (21–23). A widely used regular sequence motif for the prediction of potential G-quadruplex-forming sequences (pG4) is G_3+_N_1–7_G_3+_N_1–7_G_3+_N_1–7_G_3+_, where tracts of three or more continuous guanines will form G-quadruplex columns and linker sequences will form the connecting loops (24,25). As many as 376 000 G-quadruplex-forming sequences have been reported to exist in the human genome using this algorithm (24,25). However, it is now evident that non-canonical G-quadruplex-forming sequence motifs exist that include bulges (26–28), G-triads (29), long connecting loops (30,31), structured loops (32–35), left-handed (36) and GAGA/GCGC-tetrad containing quadruplexes (37). G4-seq has recently identified 716 310 G-quadruplex-forming sequences in the human genome, among which 451 646 sequences were non-canonical including those containing long loops and bulges (11) not predicted by previous bioinformatics searches using regular pG4 motifs (24,25).

In this study, G-quadruplex structure formation in the *PARP1* promoter DNA sequence TGGGGGCCGAGGCGGGGCTTGGG (termed as *TP3*) located 125 nucleotide (nt) upstream of the transcription start site (TSS) is examined by NMR spectroscopy. *TP3* was chosen among other sequences identified in the *PARP1* gene due the fact that it displays a well-resolved NMR spectrum which is indicative of a well-defined structure, further facilitating structural determination (Supplementary Figure S1). Our study shows the formation of a (3+1) hybrid, intramolecular, three-layered G-quadruplex topology with unique structural features.

## MATERIALS AND METHODS

### Sample preparation

Unlabeled and site-specific labelled DNA oligonucleotides were chemically synthesized on an ABI 394 DNA synthesizer using products from Glen Research and Cambridge Isotope Laboratories and then purified following the protocol from Glen Research. The samples (concentration, 0.2−2 mM) were dialyzed successively against water, 25 mM KCl, and water again. DNA oligonucleotides were frozen, lyophilized and dissolved in buffer containing 70 mM KCl and 20 mM KPi (pH 7) buffer. The DNA concentration was expressed in strand molarity using the nearest neighbor approximation for the 260 nm molar extinction coefficient of the unfolded species.

### CD/UV melting experiments

The stability of G-quadruplexes was measured via CD/UV melting experiments conducted on a JASCO V-650 spectrophotometer or a JASCO-815 spectropolarimeter. Experiments were performed with 1-cm path length quartz cuvettes. The absorbance at 295 nm was recorded as a function of temperature ranging from 15 to 95°C. Heating and cooling were done at a rate of 0.2°C/min. The DNA concentration ranged from 4 to 6 μM and experiments were carried out in buffer containing 70 mM KCl, 20 mM KPi (pH 7), as well as other buffer conditions in which the concentration of KCl and KPi were varied and KCl was substituted with LiCl (data not shown). To calculate the DNA folded fraction, all UV melting curves are manually fitted for two-state folding where two baselines are drawn at low and high temperatures corresponding to completely folded and unfolded states. The difference in the *T*_m_ values from folding and unfolding was <2°C.

### Circular dichroism

Circular dichroism (CD) spectra were recorded on a JASCO-815 spectropolarimeter using 1-cm or 0.01-cm path length quartz cuvettes in a reaction volume of 480 or 30 μl, respectively, at 25°C. Scans from 220 to 320 nm were performed with a rate of 200 nm/min, 1-nm pitch, and 2-nm bandwidth. DNA samples with concentrations of approximately 6 μM or 250 μM, dissolved in a buffer containing 70 mM KCl, 20 mM KPi (pH 7), were used for experiments. For each measurement, an average of three scans was taken, the spectral contribution of the buffer was subtracted and data were zero-corrected at 320 nm.

### Gel electrophoresis

The molecular size of the oligonucleotides was visualized by non-denaturing gel electrophoresis. Oligonucleotides were prepared in the mentioned buffer conditions. The samples were loaded on a 20% polyacrylamide gel, supplemented with 50 mM KCl and 40% sucrose was added just before loading. The gel was viewed by UV shadowing.

### NMR spectroscopy

NMR experiments were performed on Bruker Avance spectrometers operating at 600/700/800 MHz at 25°C, unless otherwise specified. The DNA concentration for NMR experiments was typically 0.2−2.0 mM in 70 mM KCl, 20 mM KPi (pH 7) at 25°C, unless otherwise specified. Note different baseline levels of the presented *TP3* spectra, which are sensitive to the sample concentration and time after dilution (see below). Assignment of the imino protons of guanine residues was obtained by ^15^N-filtered experiments using 4% site-specific labelled samples. Assignment of aromatic protons of guanine residues was obtained using both ^2^H (100%) and ^15^N (4%) site-specific labeling. In the ^2^H (100%) site-specific labeling approach, a simple 1D NMR spectrum was recorded and compared with the reference spectrum to identify the missing peak. In the ^15^N (4%) site-specific labeling approach, a 1D ^15^N–^1^H HMQC experiment was performed to filter out proton signals not coupled with ^15^N. The two approaches are complementary: the site-specific deuterium (^2^H) labeling approach requires less sample and NMR recording time, but may suffer from spectral overlap. Assignment of thymine residues was further facilitated by site-specific T-to-U substitutions. Spectral assignments were made by carrying out through-space (NOESY) and through-bond correlation experiments (TOCSY). Spectra analyses were performed using the SpinWorks (http://home.cc.umanitoba.ca/∼wolowiec/spinworks/) and SPARKY (https://www.cgl.ucsf.edu/home/sparky/) programs.

### Structure calculations

#### NOE distance restraints

Inter-proton distances for *TP3-T6* were obtained from NOESY experiments performed in H_2_O and D_2_O at various mixing times (100, 250 and 300 ms). For non-exchangeable protons, the peaks were classified as strong, medium and weak corresponding to the distance restraints of (2.7 ± 0.8), (3.8 ± 0.9) and (5.5 ± 1.7) Å respectively. Distances from exchangeable protons were classified as strong, medium and weak corresponding to the distance restraints of (3.6 ± 0.9), (4.8 ± 1.2) and (5.5 ± 1.7) Å respectively.

#### Dihedral restraints

Dihedral angle restraints were imposed to the dihedral angle formed by O4′–C1′–N9–C4 of guanine residues. *Anti* guanine residues were restricted to an angle of (240 ± 70)° or (240 ± 40)° for the outer tetrad and inner tetrad guanines respectively. *Syn* guanine residues were restricted to an angle of (60 ± 70)° or (60 ± 40)° for the outer tetrad and inner tetrad guanines respectively.

#### Hydrogen bond restraints

Hoogsteen hydrogen bonds between guanines were restrained using H21–N7, N2–N7, H1–O6 and N1–O6 distances, which were set to (2.0 ± 0.2), (2.9 ± 0.3), (2.0 ± 0.2) and (2.9 ± 0.3) Å respectively. The hydrogen bond restraints from the two interacting guanines G14 and G2 were (2.0 ± 0.2) for G14(H21)–G2(O6) and G14(H1)–G2(N7), as well as (2.9 ± 0.3) Å for G14(N2)-G2(O6) and G14(N1)-G2(N7). The hydrogen bond from G2(H1) was assigned ambiguously towards either T20(O2), T20(O4) or T20(O4′) with restraints of (2.0 ± 0.2) Å.

#### Planarity restraints

Planarity restraints were used for the G3–G21–G15–G12, G4–G11–G16–G22 and G5–G9–G17–G23 tetrads and G14–G2 base pair.

#### Repulsion restraints

Repulsion restraints between T(20)–H7 to G(14)–H1 and G(2)–H1 of (>3.5 Å) were imposed following the non-existence of the corresponding cross-peaks in the H2O NOESY spectra (both in 10°C and 25°C).

#### Distance geometry simulated annealing

An initial extended conformation of *TP3-T6* was generated using the XPLOR-NIH program (38). The system was then subjected to distance geometry simulated annealing by incorporating distance, dihedral, hydrogen bond, planarity and repulsion restraints. One hundred structures were generated and subjected to further refinement.

#### Distance-restrained molecular dynamics refinement

The 100 structures obtained from the simulated annealing step were refined with a distance-restrained molecular dynamics protocol incorporating all distance restraints. The system was heated from 300 to 1000 K in 14 ps and allowed to equilibrate for 6 ps, during which force constants for the distance restraints were kept at 2 kcal.mol^−1^.Å^−2^. The force constants for non-exchangeable proton and exchangeable proton restraints were then increased to 16 and 8 kcal.mol^−1^.Å^−2^ respectively in 20 ps before another equilibration at 1000 K for 50 ps. Next, the system was cooled down to 300 K in 42 ps, after which an equilibration was performed for 18 ps. Coordinates of the molecule were saved every 0.5 ps during the last 10.0 ps and averaged. The average structure obtained was then subjected to minimization until the gradient of energy was less than 0.1 kcal.mol^−1^. Dihedral (50 kcal.mol^−1^.rad^−2^) and planarity (1 kcal.mol^−1^.Å^−2^ for tetrads) restraints were maintained throughout the course of refinement. Ten-lowest energy structures were generated.

### Data deposition

The coordinates for the ten lowest-energy structures of the *TP3-T6* G-quadruplex have been deposited in the Protein Data Bank (PDB ID: 6AC7).

## RESULTS AND DISCUSSION

### *TP3* forms a (3+1) intramolecular G-quadruplex structure

**Figure 1.**
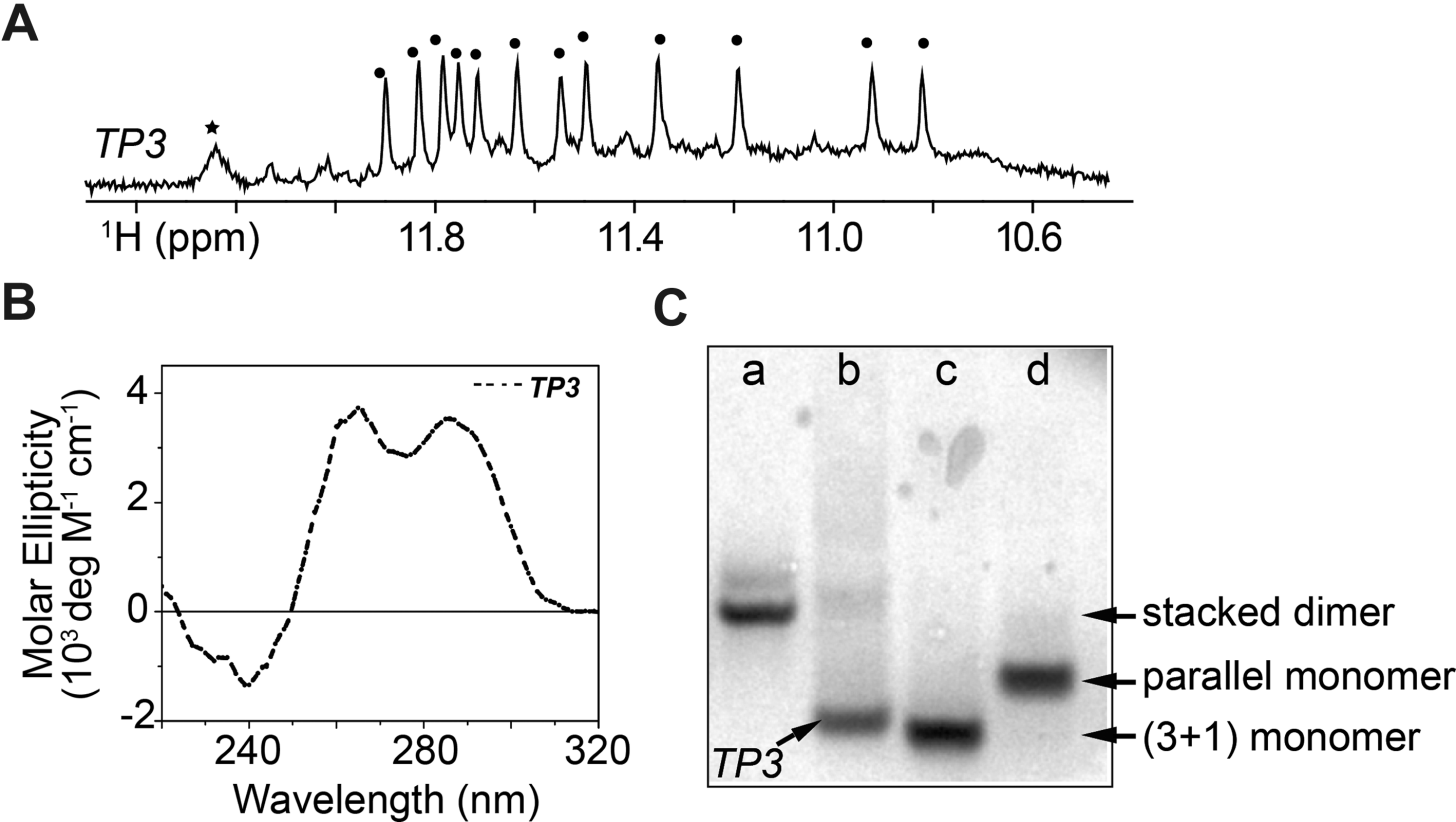
(**A**) Imino proton spectrum of *TP3*. (**B**) CD spectrum of *TP3*. (**C**) Native PAGE mobility shift analysis of *TP3* (lane-b) with other references; (lane-a) *T95* (d(GGGTGGGTGGGTGGGT), stacked dimer G-quadruplex), (lane-c) *HT* (d(TTGGGTTAGGGTTAGGGTTAGGGA), three-layer, monomer (3+1) hybrid G-quadruplex) and (lane-d) *T95-2T* (d(TTGGGTGGGTG GGTGGGT), three-layer, monomer, propeller-type parallel G-quadruplex).

*TP3* shows twelve major sharp imino proton peaks from 10.8 to 12.0 ppm (Figure 1), indicative of the formation of a major G-quadruplex fold containing three G-tetrads. An additional major peak was observed at ∼12.2 ppm (see further discussion below).

**Figure 2.**
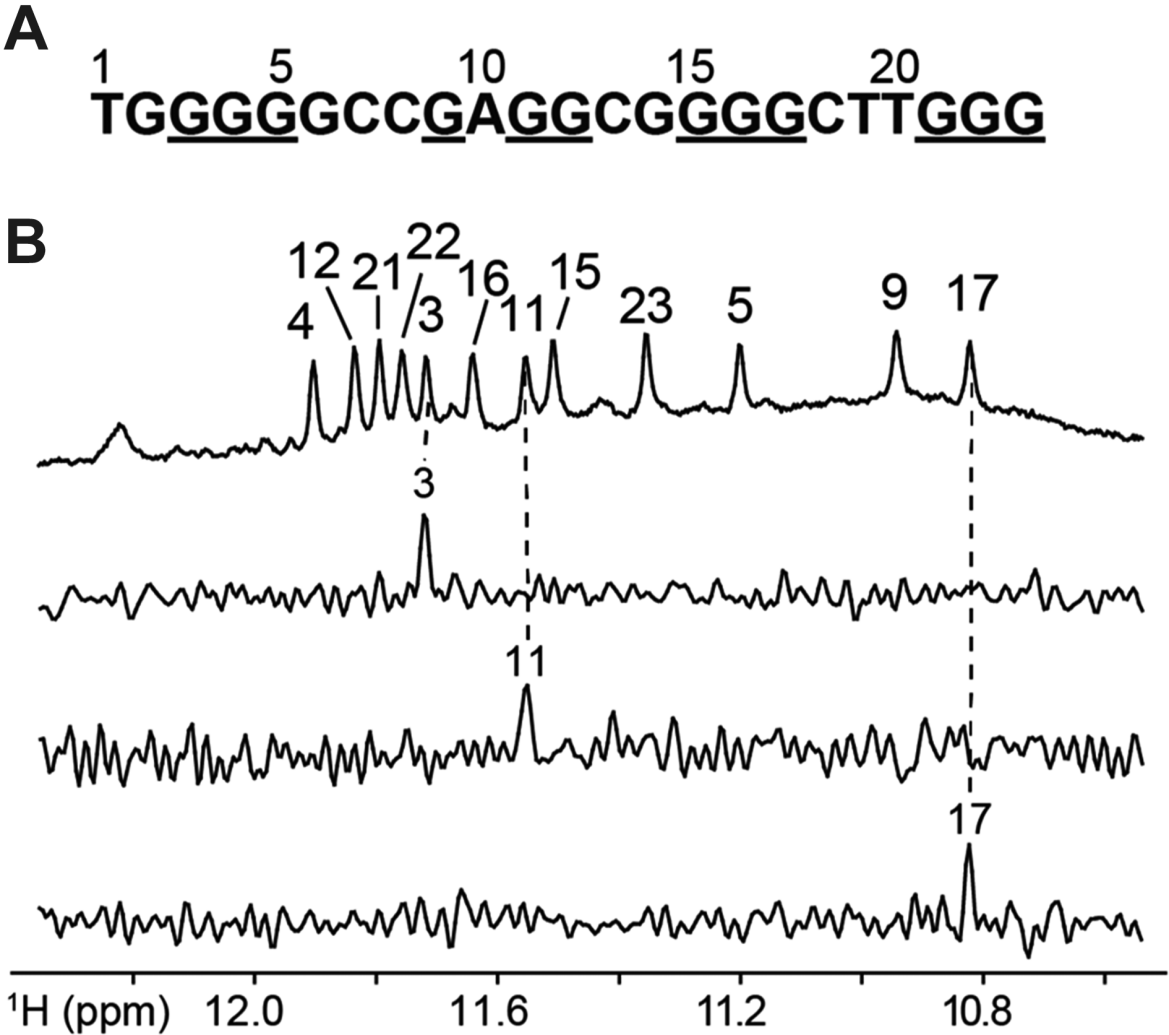
(**A**) *TP3* DNA sequence with the guanines forming G-tetrads underlined. (**B**) Imino proton resonance assignment of *TP3* indicated on the reference spectrum (top). Below are some examples of the spectral assignment of individual guanine imino protons using samples with 4% ^15^N-labeled guanines at indicated positions.

Unambiguous assignments of guanine imino (H1) and aromatic (H8) protons for the major species of *TP3* (Supplementary Figures S2 and S3) were carried out via site-specific 4% ^15^N-labeling and site-specific ^2^H substitution approaches; some representative imino proton spectral assignments are shown in Figure 2 (39). Imino proton assignments revealed the twelve guanines that are involved in the formation of the G-tetrad core (underlined, Figure 2A). The CD spectrum of the *TP3* G-quadruplex displays two positive peaks at 265 and 290 nm along with a trough at 240 nm, as previously reported for (3+1) hybrid G-quadruplexes (40–42). Native PAGE analysis compares the migration of the *TP3* G-quadruplex structure with respect to other reference G-quadruplex structures. The migration of *TP3* is comparable to that of the human telomeric (*HT*) sequence forming a three-layered, monomeric (3+1) hybrid G-quadruplex (43) and faster than other reference samples *T95-2T* (three-layered, monomeric and propeller-type parallel G-quadruplex) and *T95* (stacked dimer parallel G-quadruplex) (44). Altogether, these data suggest that *TP3* forms a three-layered monomeric G-quadruplex.

However, due to poor long-term stability of the G-quadruplex structure in *TP3* at room temperature (Supplementary Figure S4), we could not execute long two-dimensional NMR experiments to determine the structure of the *TP3* G-quadruplex. Temporal instability of the G-quadruplex fold in the *TP3* sequence could probably arise due to the formation of higher-order aggregate structures over time, as indicated by the appearance of a background hump in the imino proton NMR spectra, enhanced 265-nm peak in the CD profile and low-mobility smear bands in native PAGE (Figure 1 and Supplementary Figure S4). Despite its poor temporal stability, the *TP3* monomer displays a relatively high melting temperature (*T*_m_) of 59.6°C as evidenced by UV and CD melting experiments in the presence of 70 mM KCl and 20 mM KPi (Supplementary Figure S5). Moreover, within the transcription factor binding timescale of few milliseconds to hundred seconds (45,46), this G-quadruplex structure largely remains a monomer under physiological conditions and evolves into aggregated higher-order structures only over time.

### A small sequence change facilitated NMR structure determination

**Figure 3.**
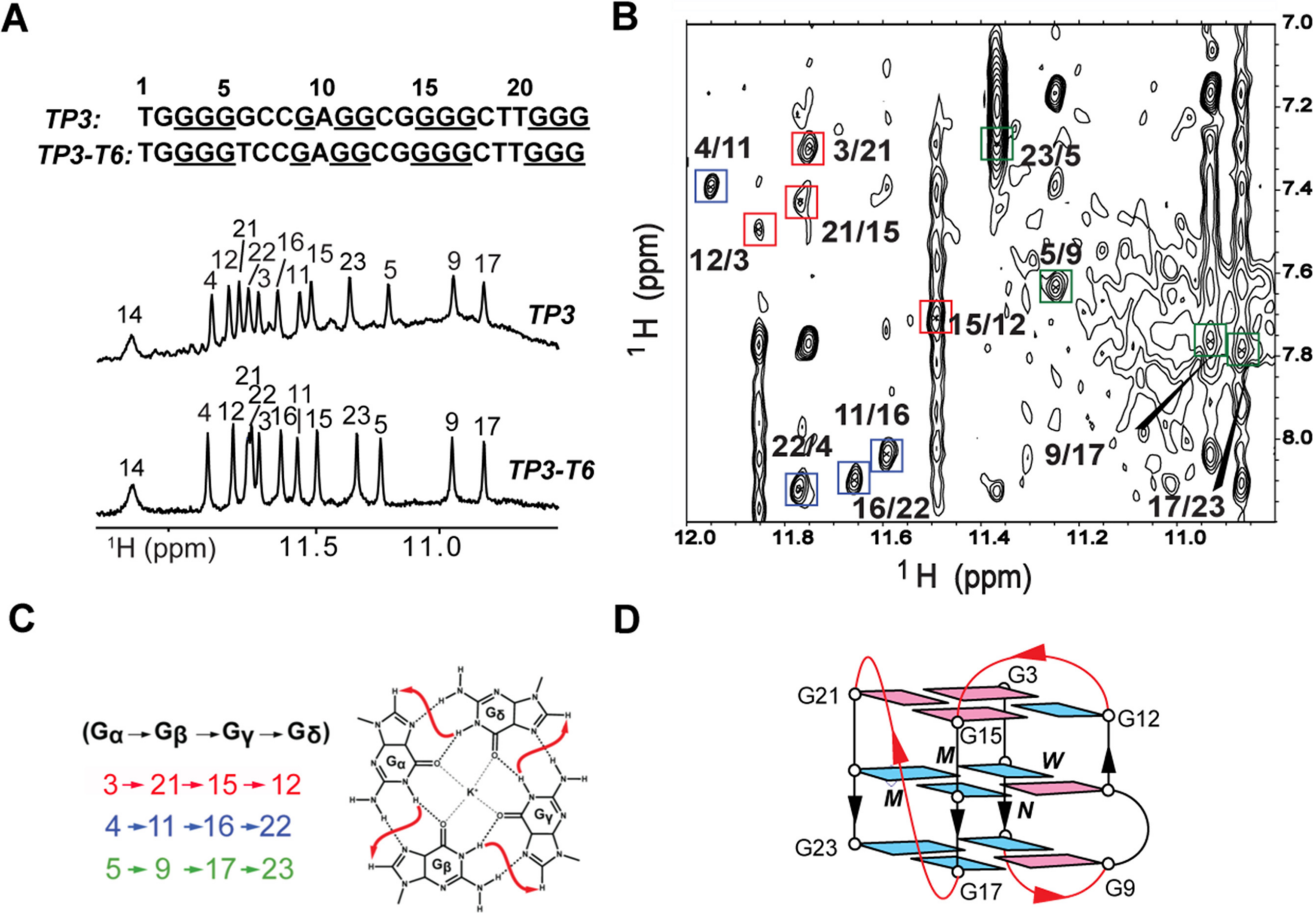
(**A**) Imino proton spectra of *TP3* and the mutated *TP3-T6* sequence with peak assignments on top. (**B**) NOESY spectrum (mixing time, 250 ms) showing the H1/H8 connectivity of G-tetrads. Guanine H1/H8 cross-peaks for G-tetrads are framed and labeled. The residues in different G-tetrads are indicated by different colors. (**C**) Specific H1-H8 connectivity pattern of the three tetrads. (**D**) Schematic of the folding topology of *TP3-T6*. *Syn* and *anti* guanines are colored as magenta and cyan respectively. *W, M* and *N* represent wide, medium and narrow groove widths respectively.

Variants of the *TP3* sequence were examined, where each non-tetrad forming guanine residue (G2, G6 and G14) was replaced by a thymine (G-to-T substitution). Imino proton spectra of mutants show that single thymine mutations at G2 and G14 result in the co-existence of multiple G-quadruplexes (Supplementary Figure S6), while G-to-T substitution at position 6 (*TP3-T6*) shows a NMR spectrum very similar to that of *TP3* (Figure 3A), indicating the formation of the same G-quadruplex fold. Furthermore, the *TP3-T6* G-quadruplex structure displays improved temporal stability upon exposure to room temperature, enabling a detailed NMR structural study. CD spectra of *TP3-T6*, measured at concentrations of 6 and 250 μM, displays two positive peaks at 265 and 290 nm along with a trough at 240 nm (Supplementary Figure S7), indicative of a (3+1) hybrid G-quadruplex, as observed for *TP3*.

We proceeded to study the G-quadruplex structure of the *TP3-T6* sequence in more detail. Spectral assignment of *TP3-T6* was carried out by the site-specific (4%) ^15^N-enrichment approach. Guanine imino (H1) and aromatic (H8) protons were unambiguously assigned for *TP3-T6* (Supplementary Figure S8) and some of the assignments were also extrapolated from *TP3* (Supplementary Figures S2 and S3) and vice versa as proton chemical shifts and spectral patterns in both sequences are extremely similar. The imino proton at ∼12.2 ppm in *TP3-T6* was assigned to G14 by site-specific ^15^N-enrichment experiments (Supplementary Figure S8). The imino proton of G2 was identified at ∼10.8 ppm by ^15^N-enrichment experiments at 10°C (Supplementary Figure S9). Assignments of thymine residues were determined via through-bond correlation experiments as well as T-to-U substitutions (Supplementary Figure S10). The folding topology was determined using NOESY experiments (Figure 3 and Supplementary Figures S11 and S12). Cyclic NOE connectivities between the imino (H1) proton of a guanine and the aromatic (H8) proton of the neighboring guanine in the same tetrad (Figure 3B) established G-tetrad alignments for *TP3-T6*: G3·G21·G15·G12 (red), G4·G11·G16·G22 (blue) and G5·G9·G17·G23 (green) (Figure 3C). High-intensity intra-residue H8-H1′ NOE cross peaks were observed for five guanine residues G3, G9, G11, G15 and G21 (Supplementary Figure S11), indicating the *syn* glycosylic conformation adopted by them, whereas other guanines in the tetrad core adopt an *anti* glycosylic conformation. NOE sequential connectivity between residues H1′(*n*)-H8(*n*+1) strongly depends on their glycosylic conformation (39). *Syn*-to-*anti* inter-residue connections, are supported by the observation of specific rectangular NOE patterns of H1′(*n*)–H8(*n*+1) and H1′(*n*+1)–H8(*n*) cross peaks (Supplementary Figure S11). Additionally, *syn·syn·syn·anti* alignment of the top and *anti·anti·anti·syn* alignment of the two bottom G-tetrads are confirmed by guanine H1–H1 and H8–H8 NOE peaks (Supplementary Figure S12). Thus, the folding topology of *TP3-T6* reveals a (3+1) hybrid G-quadruplex scaffold, in which three strands (involving the G3–G5, G15–G17 and G21–G23 tracts) are oriented in one direction and the fourth (G9–G12 tract) in the opposite direction connected by two edgewise loops and one propeller loop (Figure 3D). Strand orientations in *TP3-T6* result in two medium grooves, one narrow groove and a wide groove being present. The first edgewise loop (TCC) connects across the wide groove at the bottom, the second edgewise loop (CG) connects across the narrow groove at the top, while the third propeller loop (CTT) traverses across the medium groove connecting two parallel stands.

G4, G11, G16 and G22 form the central tetrad layer. This is independently confirmed in solvent exchange experiments, whereby the imino protons of these residues are among the most protected after 20 minutes (Supplementary Figure S13). Adenine interruption (A10) in the second guanine tract is incorporated as a bulge between two tetrads. The close proximity of G11 to the A10 bulge might account for the relatively shorter exchange time of the G4 imino proton, which is hydrogen-bonded to G11(N7), as compared to other central guanines.

### Solution structure of the *TP3-T6* quadruplex

The solution structure of *TP3-T6* was computed based on NMR restraints (Table 1) extracted from NOE cross-peaks in NOESY experiments.

**Table 1.** Statistics of the computed structures of *TP3-T6*^a^

A. NMR restraints	
distance restraints	
Intraresidue	
Exchangeable	0
Non-exchangeable	312
Interresidue	
Exchangeable	45
Non-exchangeable	75
other restraints	
hydrogen-bond restraints	53
dihedral restraints	13
planarity restraints	4
repulsion restraints	2
B. Structure Statistics	
NOE violations	
number (>0.2 Å)	0.2 ± 0.4
deviations from the ideal covalent geometry	
bond lengths (Å)	0.003 ± 0.000
bond angles (deg)	0.687 ± 0.006
impropers (deg)	0.339 ± 0.005
pairwise all heavy atom rmsd values (Å)	
G-tetrad core	0.402 ± 0.122
all residues	0.601 ± 0.142

^a^PDB ID 6AC7.

**Figure 4.**
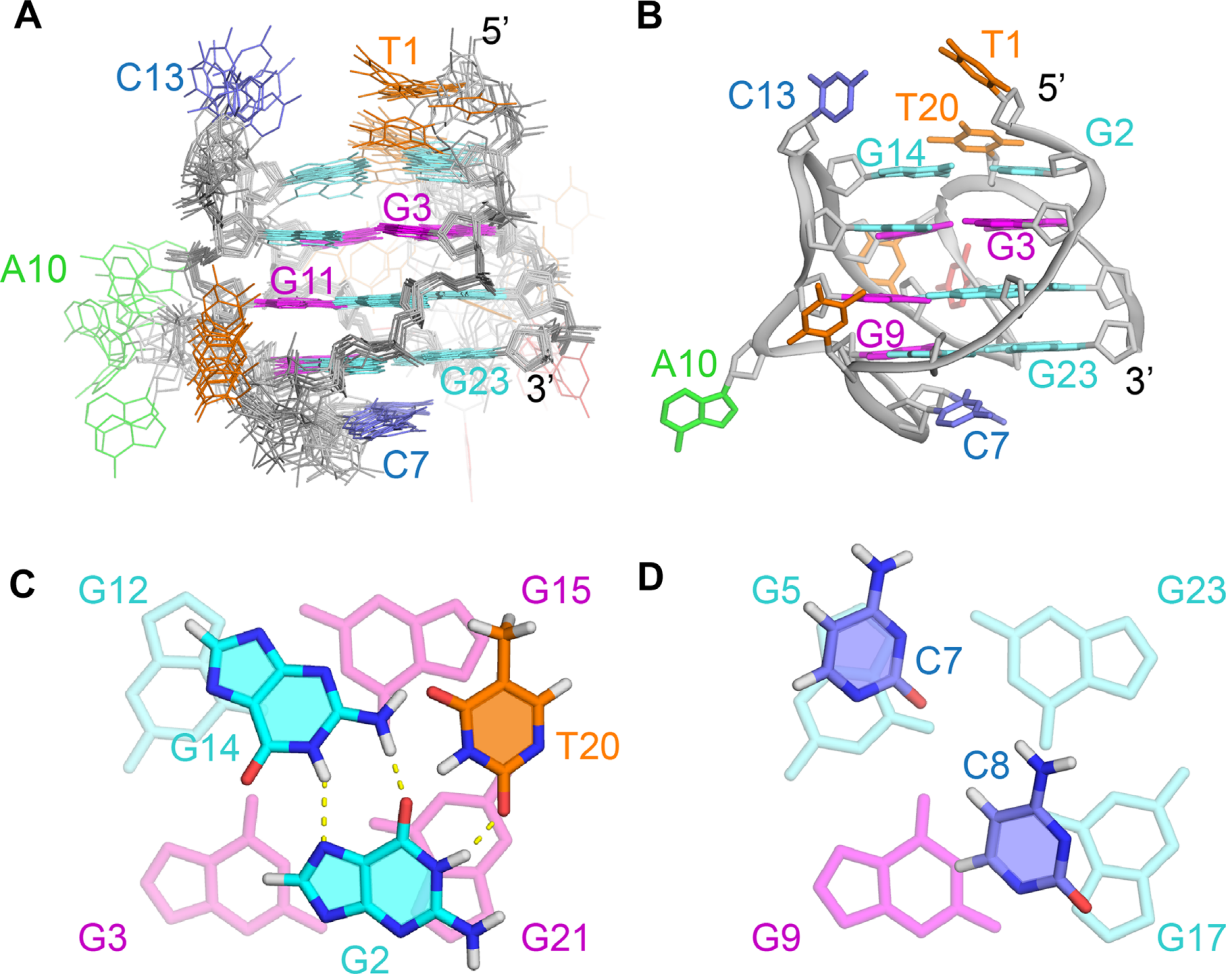
NMR solution structure of the *TP3-T6* G-quadruplex (PDB ID: 6AC7). (**A**) Ten lowest energy superimposed refined structures. (**B**) Ribbon view of a representative structure. *Anti* guanine residues are colored cyan, *syn* guanine residues are colored magenta, thymine residues are colored orange, cytosine residues are colored red and adenine residues are colored green. (**C**) Close-up view of the G2·G14·T20 base triple capping the top of the structure. (**D**) Close-up view of the C7 and C8 bases at the bottom of the structure.

The ten lowest-energy structures are presented in Figure 4A. The G-tetrad core displays a greater convergence compared to the loops. Stacking interactions are observed between cytosine residues C7 and C8 in the TCC edgewise loop and guanine residues in the bottom tetrad (Figures 4).

### Formation of a base triple capping structure

**Figure 5.**
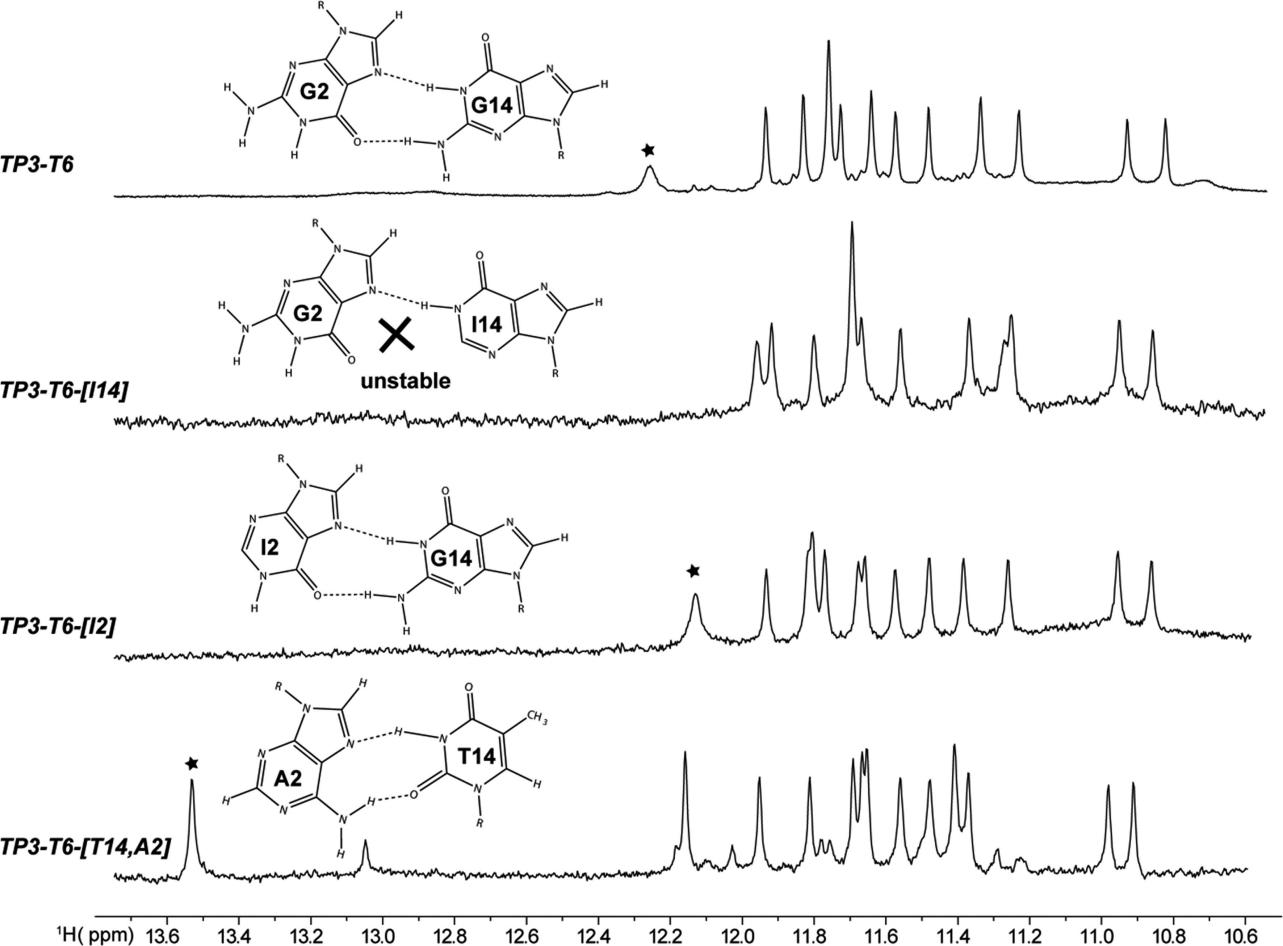
Imino proton spectra of *TP3-T6* and sequences mutated at the indicated positions. An asterisk indicates the imino proton in the Hoogsteen base pair between residues 2 and 14.

In addition to the three G-tetrad layers, the structure also possesses a G2·G14·T20 base triple (Figure 4C), where the three bases come from distinct strands. G2 and G14 interact via reverse Hoogsteen bonding, with H21 and H1 of G14 hydrogen bonding with O6 and N7 of G2 respectively. This is further supported by the observation of a NOE peak between the aromatic (H8) proton of G2 with the imino (H1) proton of G14 (Supplementary Figure S14). Inosine-substitution experiments at G2 and G14 positions also confirm the reverse Hoogsteen hydrogen bonding between G2 and G14, where the G14 imino proton peak vanishes for I14 substitution but remains for the I2 substitution (Figure 5). We also observed that the reversed Hoogsteen base pair G14·G2 could be substituted by an alternative Hoogsteen base pair T14·A2 (Figure 5).

The capping structure confers additional stability to *TP3-T6* and a higher thermal stability is observed for *TP3-T6* as compared to *TP3-T6-[I14]* in which the capping structure is disrupted (Supplementary Figure S15 and Table 2). Such hydrogen-bond interactions between edgewise loop residues and 5′ terminal residues were previously reported in the literature for (3+1) hybrid G-quadruplexes, where they have been found to play a critical role in the stabilization of one conformation over the other by providing additional stability to the structure (43,47–52).

**Table 2.** Thermal stability of *TP3-T6* and mutated sequences

Name	Sequence (5′ → 3′)	*T*_m_ (°C)^a^
*TP3-T6*	TGGGGTCCGAGGCGGGGCTTGGG	60.2 ± 0.9
*TP3-T6-[I14]*	TGGGGTCCGAGGC**I**GGGCTTGGG	57.3 ± 0.6
*TP3-T6-[I2]*	T**I**GGGTCCGAGGCGGGGCTTGGG	60.2 ± 0.7
*TP3-T6-[T14, A2]*	T**A**GGGTCCGAGGC**T**GGGCTTGGG	63.6 ± 0.8

^a^Melting temperature (*T_m_*) was measured in 70 mM KCl and 20 mM KPi (pH 7).

Addtionally, we detected a sharp imino proton resonance of G2 at 10°C (Supplementary Figure S9), indicative of the hydrogen bonding present between G2 and T20 (Figure 4C and D). Therefore, the overall base triple capping structure in *TP3-T6* comprises of the 5′ terminal G2 residue which is centrally positioned and forms hydrogen bonding with G14 in the edgewise loop and T20 in the propeller loop. The resulting G·G·T capping structure formed on the top tetrad serves as a protective layer, limiting the exposure of guanine residues of the top tetrad to solvent, which is in agreement with solvent exchange experiments whereby the imino protons of G12 and G15 located in the top tetrad are protected from D_2_O solvent exchange after 20 minutes and the imino proton of G15 remains protected even after 2 h (Supplementary Figure S13). Similar base triple capping structures stacked on the top and bottom tetrad, such as A·A·A, T·G·A, T·A·T, T·T·T and protonated T18·A20^+^·G5 base triples have been previously reported to contribute to the overall stability of the quadruplex or favor one particular conformation over another (47–55).

### Bulge-containing (3+1) hybrid G-quadruplex scaffold for drug targeting

Research over the past 25 years on G-quadruplexes has resulted in the discovery of several G-quadruplexes with a (3+1) G-tetrad core, in which three strands are oriented in one direction and the fourth in the opposite direction. The (3+1) hybrid folding topology was first observed for a G-quadruplex formed by the *Tetrahymena* telomeric sequence d(T_2_G_4_)_4_ in Na^+^ solution, whereby a three-layered intramolecular G-quadruplex with a *syn·syn·syn·anti* G-tetrad and two *anti*·*anti*·*anti*·*syn* G-tetrads was reported (56). A dimeric, asymmetric quadruplex in Na^+^ solution formed by the human telomeric sequence d(GGGTTAGGGTTAGGGT), as well as intramolecular G-quadruplexes in K^+^ solution formed by the human telomeric sequence d[TAGGG(TTAGGG)_3_] and many variants, were also later found to adopt a (3+1) hybrid folding topology (43,49–52,57). Besides the telomeric region, (3+1) hybrid G-quadruplexes have also been found to be present in several gene promoters such as *hTERT, BCL2* and *VEGFR-2*, highlighting a possible role for such scaffolds in transcriptional regulation (47,58,59). In this study, we have revealed a (3+1) hybrid G-quadruplex in the promoter of *PARP1*—a gene with a very important role in DNA repair and of significant therapeutic relevance.

Besides being characterized by loop and strand orientations, G-quadruplexes can also be characterized by unique and intrinsic structural features such as bulges, providing recognition elements that can, in principle, be exploited for specific small-molecule targeting. The bulge formation was observed in a variety of contexts with varying the type, the number and the positions of bulge residues, as well as the type of the G-quadruplex scaffold, suggesting a widespread existence of bulge-containing G-quadruplex-forming sequences (26). Several G-quadruplex forming sequences with residues capable of forming single-nucleotide or multi-nucleotide bulges upon folding into a G-quadruplex were identified by the G4-seq method via sequencing DNA from primary human B lymphocytes (11). Thymine bulges within parallel-stranded G-quadruplexes have been reported for *LTR-IV*, present in the long terminal repeat promoter region of HIV-1, *22RT*, present within the *KRAS* nuclease-hypersensitive element (NHE) region, and *AT11*, an anti-proliferative oligonucleotide (27,28,60). A cytosine bulge has also been observed within the parallel-stranded RNA and DNA G-quadruplexes formed by the 5′-end sequence of hTERC (61,62). Although adenine bulges have also been previously reported to exist within G-quadruplex structures (26,59), we report here the solution structure of a G-quadruplex with an adenine bulge in a new structural context. The detailed structural characteristics of the (3+1) hybrid G-quadruplex *TP3-T6* with an adenine bulge present an opportunity for PARP1 regulation by selective non-canonical G-quadruplex targeting.

## CONCLUSION

We have revealed the (3+1) hybrid G-quadruplex folding topology adopted by the G-rich promoter sequence of the *PARP1* gene. The interesting structural features of the *PARP1* G-quadruplex are an adenine residue bulge and a unique G·G·T triple capping structure formed by the central edgewise loop, propeller loop and 5′ flanking terminal which could be exploited as distinct recognition elements to generate small-molecule ligands to target this G-quadruplex structure with specificity. This structure presents an opportunity to further explore the therapeutic potential of PARP1 inhibition via selective G-quadruplex DNA targeting.

## DATA AVAILABILITY

SpinWorks and SPARKY are open source softwares available at http://home.cc.umanitoba.ca/∼wolowiec/spinworks/ and https://www.cgl.ucsf.edu/home/sparky/ respectively.

Atomic coordinates and structure factors for the reported NMR structures have been deposited with the Protein Data bank under accession number 6AC7.

## Supplementary Material

Supplementary Data
