# Supplementary material for: Structure of a (3+1) hybrid G-quadruplex in the PARP1 promoter
Source: Nucleic Acids Res. 2018 Dec 15;47(3):1564–72. doi: 10.1093/nar/gky1179 (PMC6379715; doi:10.1093/nar/gky1179)
Supplement: Supplementary Data [file gky1179_supplemental_files.pdf]

Structure of a (3+1) hybrid G-quadruplex in the *PARP1* promoter

Anjali Sengar[1#], J Jeya Vandana[1#], Vicki S Chambers[2], Marco Di Antonio[2], Fernaldo Richtia Winnerdy[1], Shankar Balasubramanian[2,3] and Anh Tuân Phan*

[1] School of Physical and Mathematical Sciences, Nanyang Technological University, Singapore 637371

[2] Department of Chemistry, University of Cambridge, CB2 1EW, UK
[3] Cancer Research UK Cambridge Institute, University of Cambridge, Li Ka Shing Centre, Cambridge CB2 0RE, UK

* To whom correspondence should be addressed. Tel: (+65) 6514 1915; Fax: (+65) 6795 7981; Email: phantuan@ntu.edu.sg
#: The authors wish it to be known that, in their opinion, the first two authors should be regarded as joint First Authors.

SUPPLEMENTARY DATA

Keywords: DNA structures · G-quadruplexes · NMR spectroscopy · PARP1· BRCA mutation

SUPPORTING INFORMATION CONTENTS

Fig S1 - G-quadruplex forming sequences identified in the *PARP1* promoter by G4-seq approach

Fig S2 - Imino proton resonance assignments of *TP3*

Fig S3 - Aromatic proton resonance assignments of *TP3*

Fig S4 - Time evolution of *TP3* monomers and higher order aggregates at room temperature

Fig S5 - Superimposition of normalized CD and UV melting curves of *TP3*

Fig S6 - Imino proton spectra region of *TP3* and mutated *TP3* sequences

Fig S7 - CD spectrum of *TP3* (6 µM) and *TP3-T6* (6 µM and 250 µM)

Fig S8 - Imino (H1) and aromatic (H8) proton assignments for the *TP3-T6* sequence

Fig S9 - Imino (H1) proton resonance for G2 of the *TP3-T6* sequence

Fig S10 - Unambiguous assignment of thymine 20 by substitution of deoxyuridine (dU) for dT

Fig S11 - NOESY spectrum of *TP3-T6* (mixing time, 300 ms).

Fig S12 - NOESY spectrum of *TP3-T6* showing H1-H1 through space correlations labeled as indicated on the spectrum and H8-H8 cross-peaks showing sequential connection

Fig S13 - Imino proton spectrum of *TP3-T6* recorded after 20 min and 2 hour exposure in D_2O

Fig S14 - NOESY spectrum of *TP3-T6* (mixing time, 250 ms) at 10°C in 90% H_2O and 10% D_2O

Fig S15 - Normalized CD melting curves of *TP3-T6* and sequences mutated at specified positions

A

Sequence Name	Sequence (5'-3')	Position from TSS
TP1	TGGGACAGAACAATCAAAGGGGTGGCGCCGG	-172 (-)
AP2	AGGGGGAGGGGTTGGGGGTAAAATTAGTTGTG	-222 (-)
TP3	TGGGGGCCGAGGCGGGGCTTGGG	-125 (+)

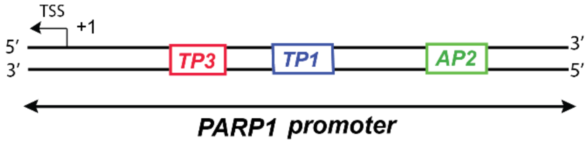

B

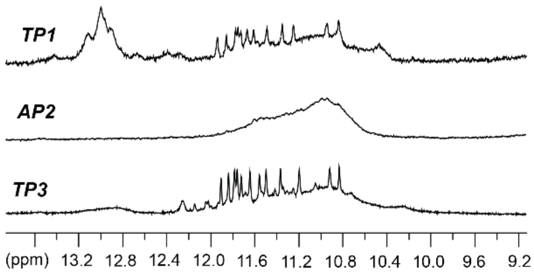

Figure S1. A. G-quadruplex forming sequences identified in the *PARP1* promoter by G4-seq approach and their respective locations from the transcription start site (TSS). The TP3 sequence, identified, is the closest to the TSS. B. ^{1}H-NMR spectra displaying the imino proton resonances of sequences identified by G4-seq

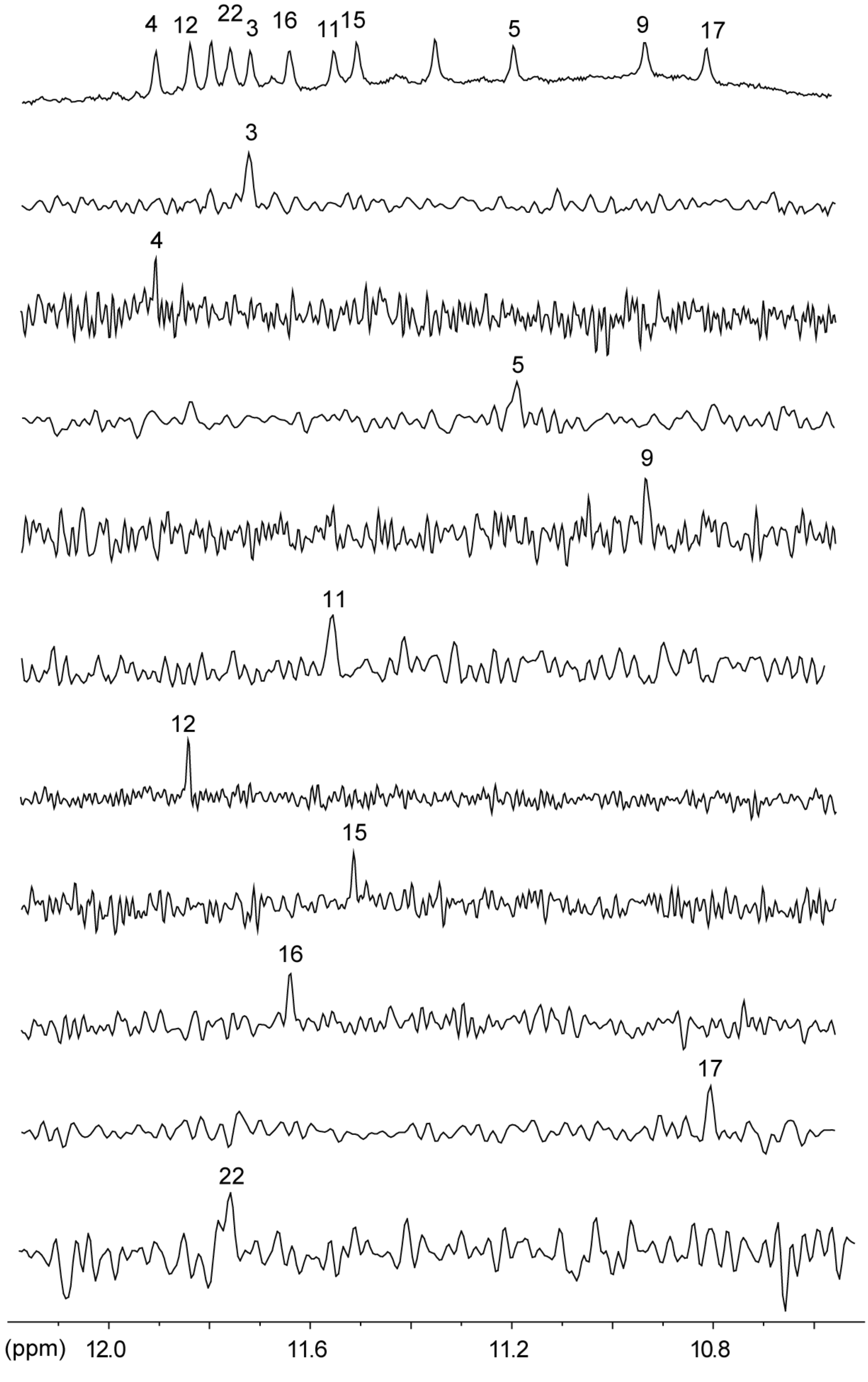

Figure S2. Imino proton resonance assignments of *TP3* indicated on the reference spectrum (top). Below are the individual spectra of 4% ^{15}N labeled guanines at indicated positions. The imino protons of G21 and G23 (which were not labelled), could be assigned by elimination and extrapolation from the assignment *TP3-T6* (see below).

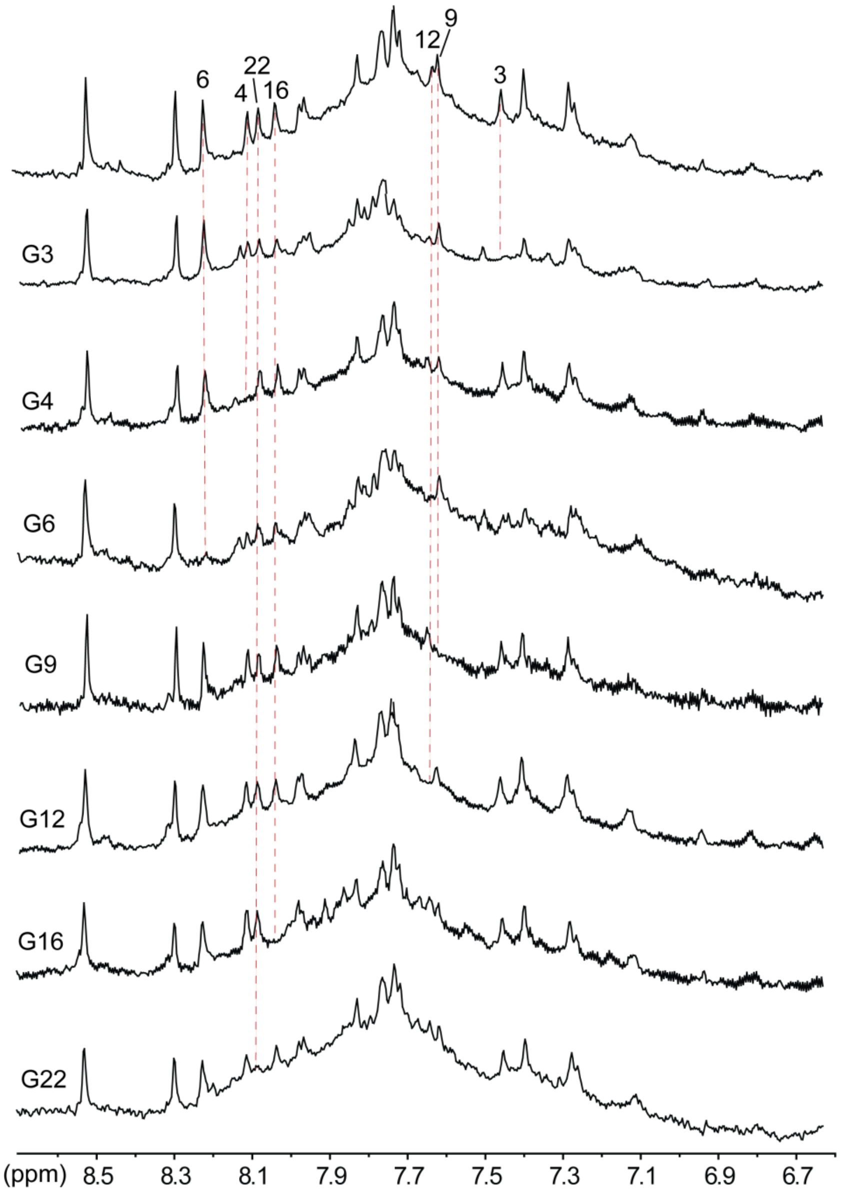

Figure S3. Aromatic proton resonance assignments of *TP3* indicated on the reference spectrum (top). Below are the individual spectra of ^{2}H-labeled guanines at indicated positions.

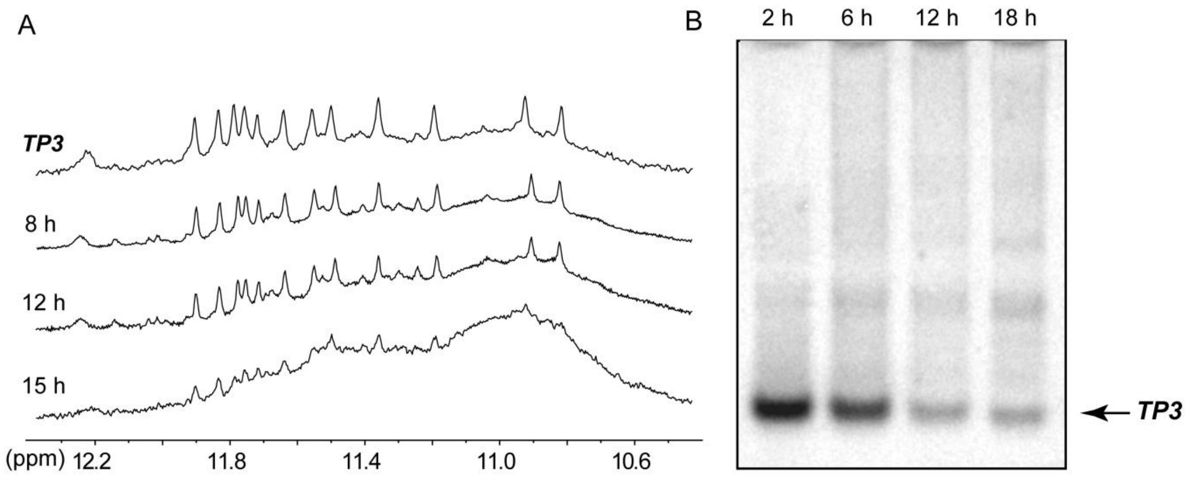

Figure S4. Time evolution of *TP3* monomers and higher order aggregates **at room temperature A. Imino proton resonances of *TP3* at varying time intervals B. Time-dependent native PAGE analysis of *TP3*.**

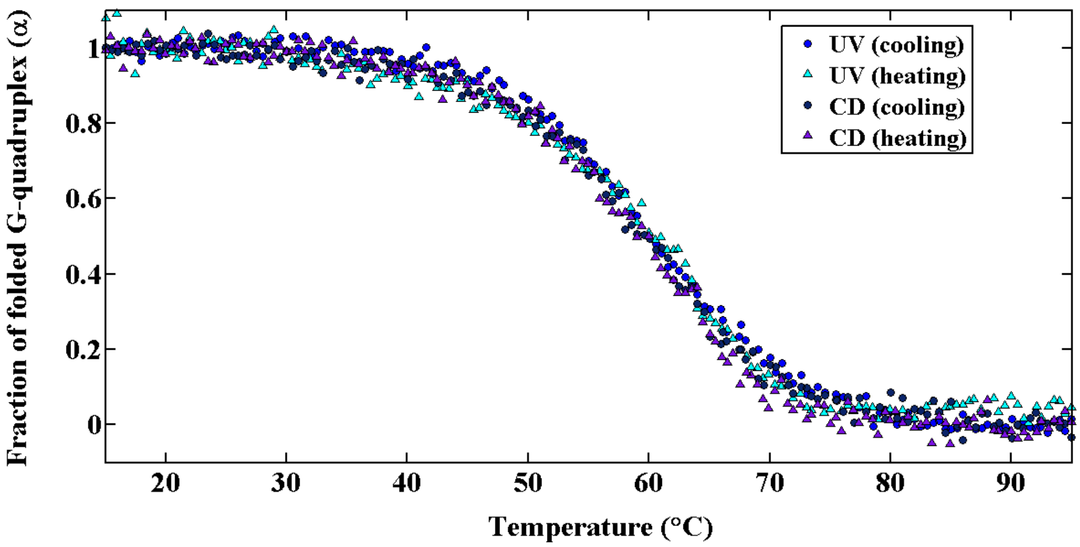

Figure S5. Superimposition of normalized CD and UV melting curves of *TP3* in 70 mM KCl and 20 mM KPi (pH = 7.0) with T_m = 59.6°C as deduced from the UV and CD melting curves. Both are comparable, with a difference of less than 1°C. The sample concentration used is ~5 uM at which TP3 predominantly remains as a monomer.

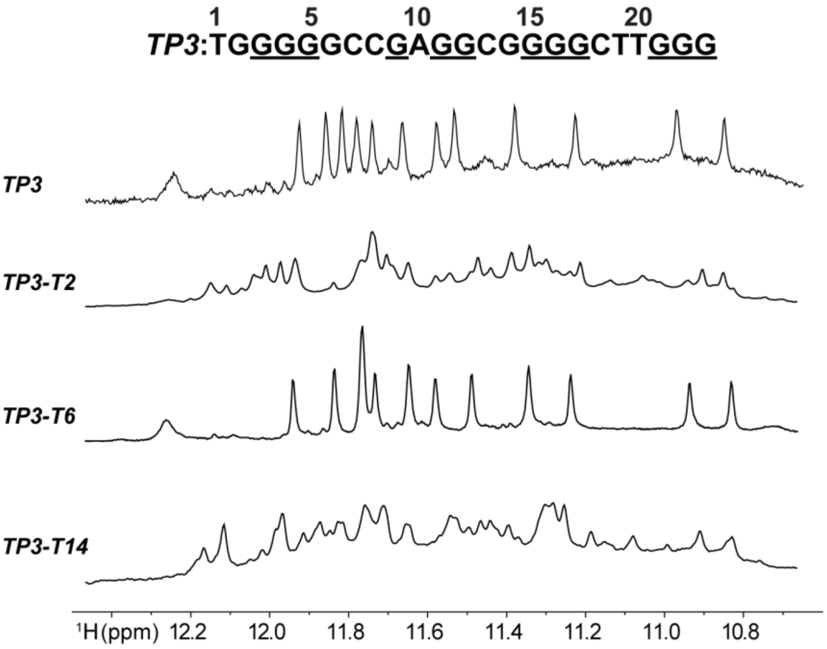

Figure S6. Imino proton spectra region of *TP3* and mutated *TP3* sequences **(G to T substitution at indicated positions).**

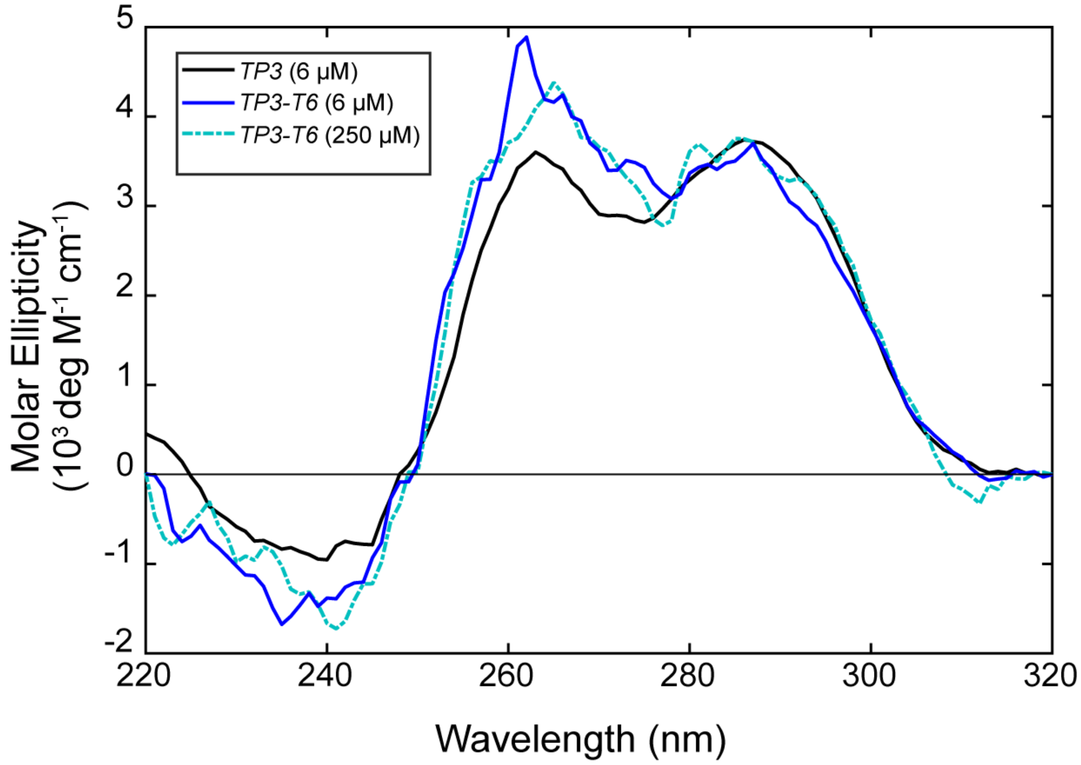

Figure S7. CD spectra of *TP3*, measured at a concentration of 6 µM, and *TP3-T6*, measured at concentrations of 6 µM and 250 µM respectively. All CD spectra display a (3+1) hybrid G-quadruplex, with 2 positive peaks at 265 and 290 nm and a trough at 240 nm in 70 mM KCl, 20 mM KPi (pH 7).

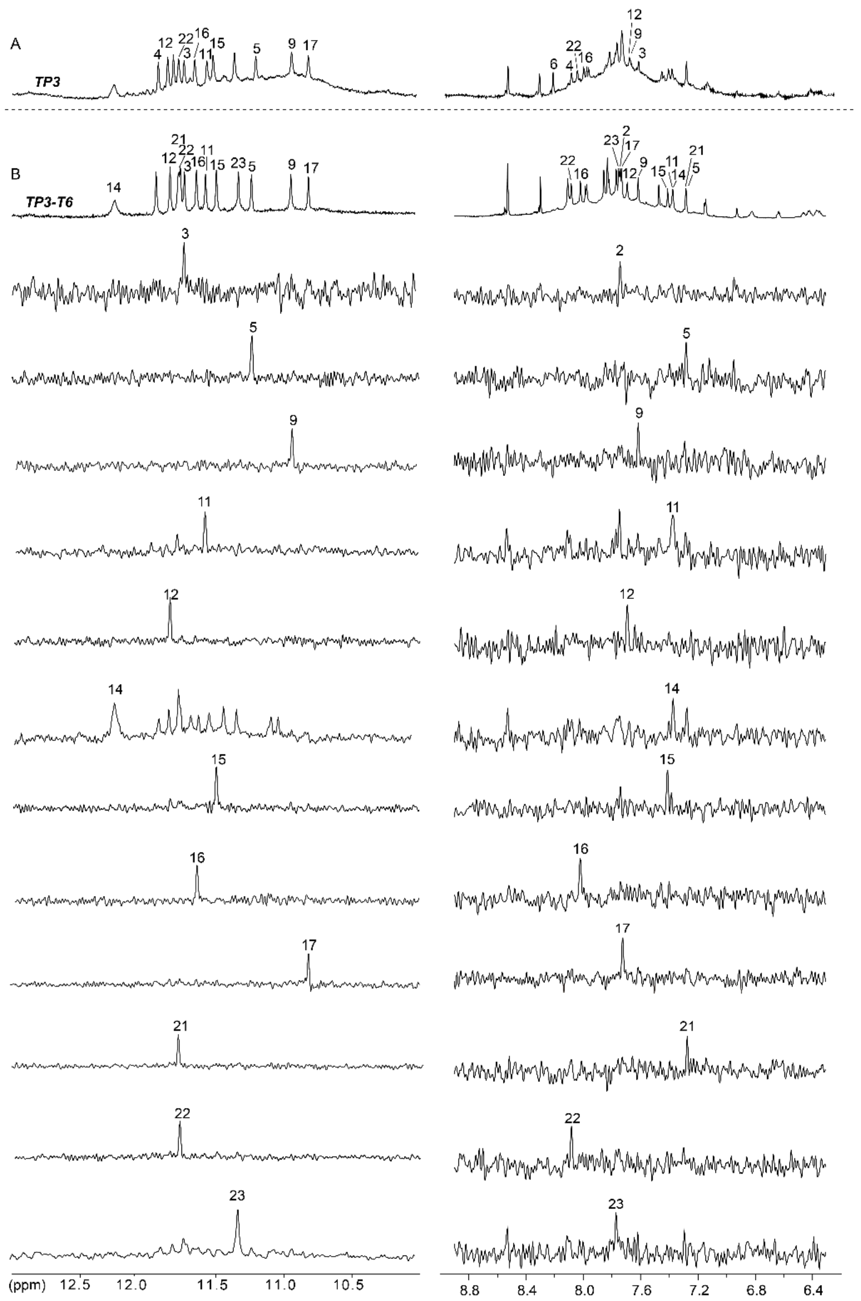

Figure S8. Imino (H1) and aromatic (H8) proton assignments for the *TP3-T6* sequence. A. Imino (H1) and aromatic (H8) proton assignments indicated on the reference spectrum for *TP3*. The imino proton of G4 (which was not labelled), could be assigned by elimination and extrapolation from the assignment *TP3.* B. Imino (H1) and aromatic (H8) proton assignments indicated on the reference spectrum on top for *TP3-T6*. Below are the individual spectra of TP3-T6 with 4% ^{15}N labeled guanine bases at indicated positions. ^{15}N-labeling was carried out for most guanine residues of TP3-T6 and TP3. Due to the high similarity of NMR spectral patterns, TP3-T6 and TP3 were deduced to have a highly similar fold and therefore, assignments were extrapolated between spectra for TP3-T6 and TP3. ^{15}N-labeling was carried out for G14 which was not part of the G-quadruplex core. Additional peaks corresponding to other guanine residues were observed for the individual spectrum of G14 due to the natural abundance of ^{15}N as a result of the accumulation of a large number of scans.

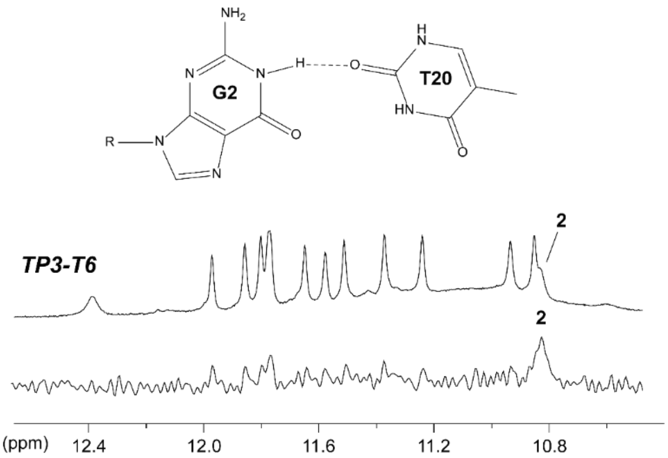

Figure S9. Imino (H1) proton resonance for G2 of the *TP3-T6* sequence resulting from the hydrogen bonding of G2 with the neighboring T20 residue as shown above. Spectra were recorded at 10°C in order to visualize the G2 peak.

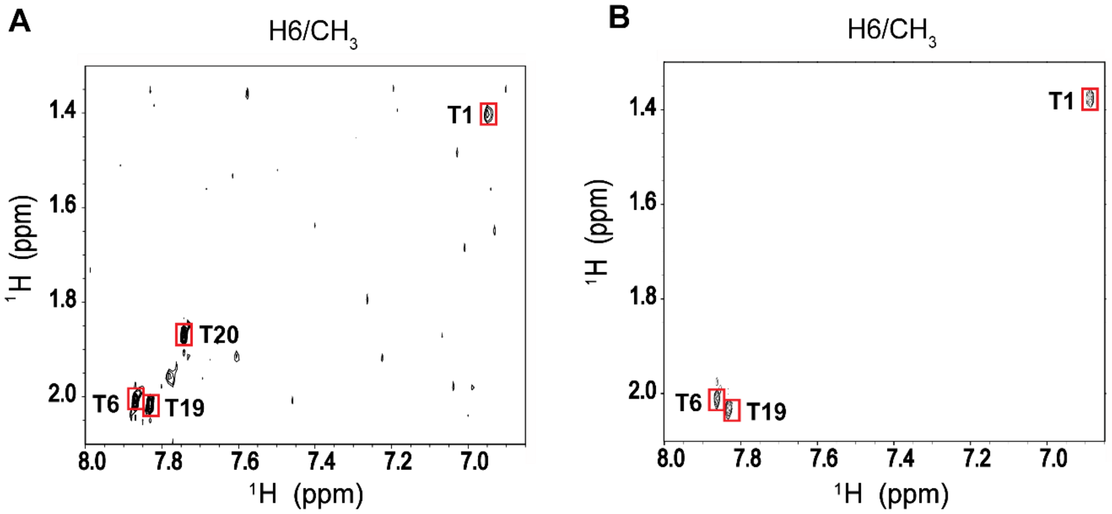

Figure S10. A. Unambiguous assignment of thymine 20 by substitution of deoxyuridine (dU) for dT at position 20 A. All four H6-CH_3 cross peaks corresponding to the four thymines in the sequence are observed in the TOCSY spectrum for *TP3-T6* B. No H6-CH_3 cross peak is observed for *TP3-T6-U20* where dU is substituted for dT at position 20.

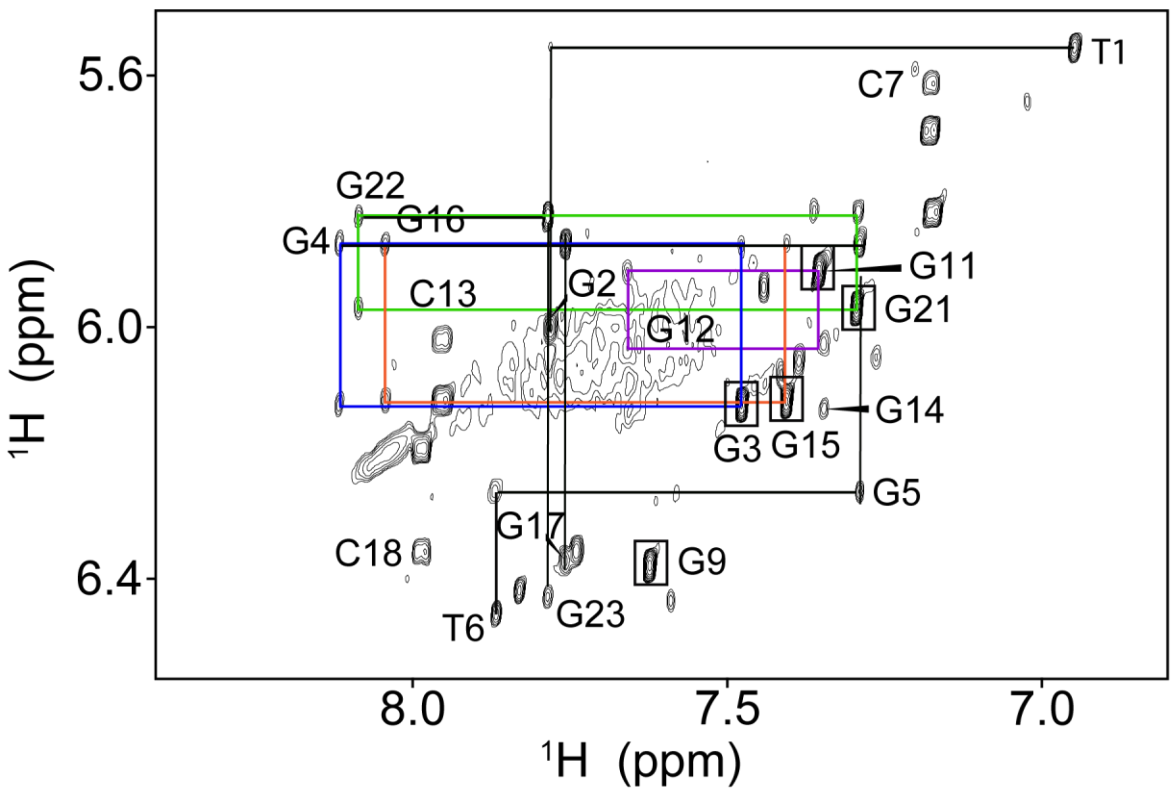

Figure S11. NOESY spectrum of *TP3-T6* (mixing time, 300 ms). Intra-residue NOE correlations are labeled according to their respective nucleotide number. Cross-peaks of strong intensity are framed in black and represent residues 3, 9, 11, 15, and 21 which adopt the *syn* glycosilic orientation. Sequential syn-to-anti (3-4, 11-12, 15-16 and 21-22) walk traced by blue, magenta, red and green rectangles respectively. Anti-to-anti connectivities that are present are also traced in black.

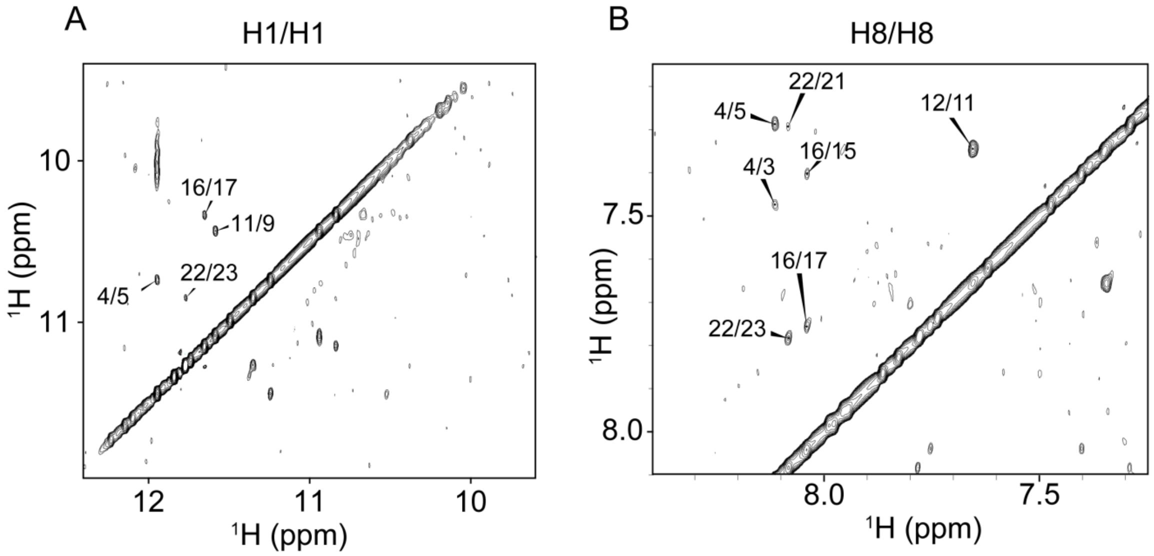

Figure S12. A. NOESY spectrum of *TP3-T6* showing H1-H1 through space correlations labeled as indicated on the spectrum. Medium intensity cross peaks for the guanine pairs (G4/G5, G9/G11, G16/G17 and G22/G23) in adjacent tetrads is observed confirming same polarity for the central and bottom tetrad (clockwise) and opposite polarity of the top tetrad (anticlockwise). B. H8-H8 cross-peaks showing sequential connection.

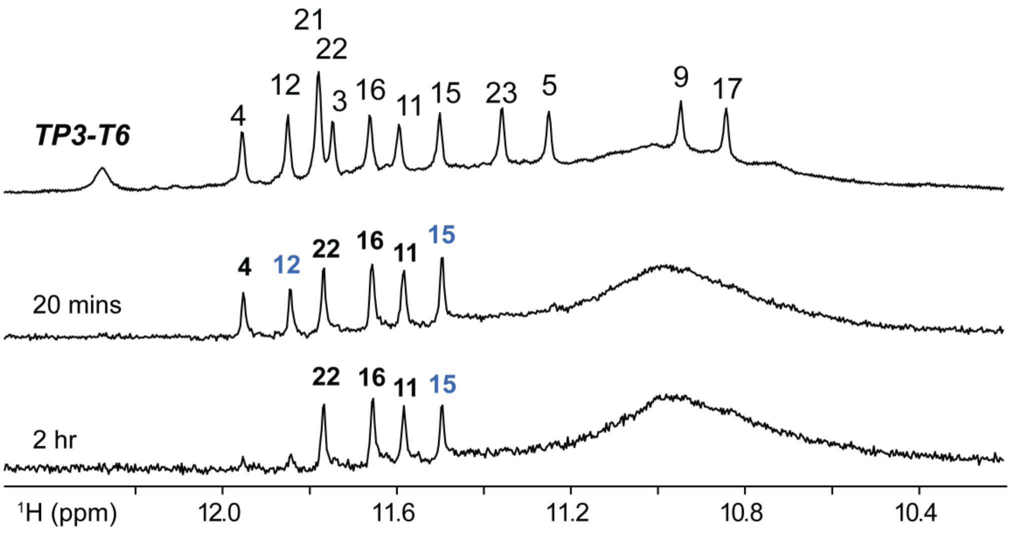

Figure S13. Imino proton spectrum of *TP3-T6* recorded after 20 min and 2 hour exposure in D_2O, showing protected residues corresponding to the middle tetrad (in black). Residues in blue are also protected and belong to the top tetrad.

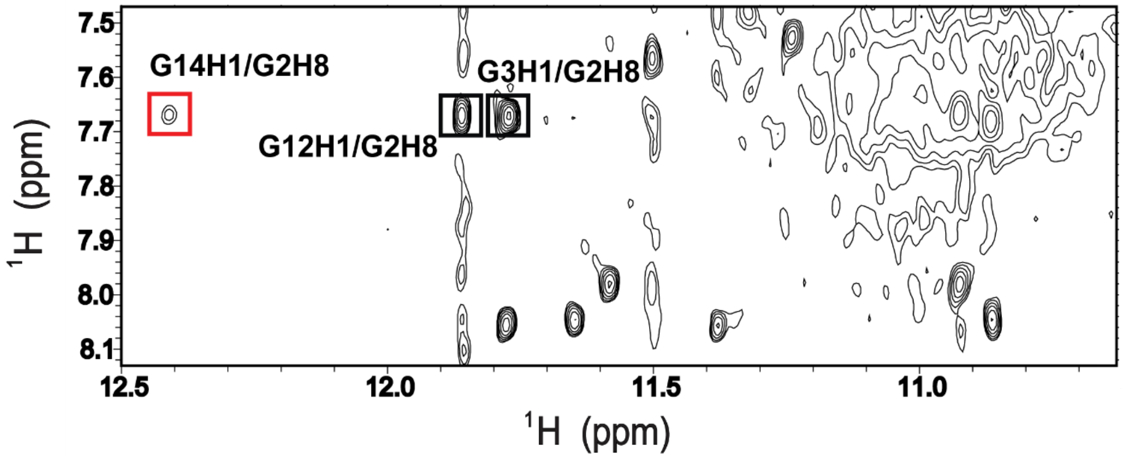

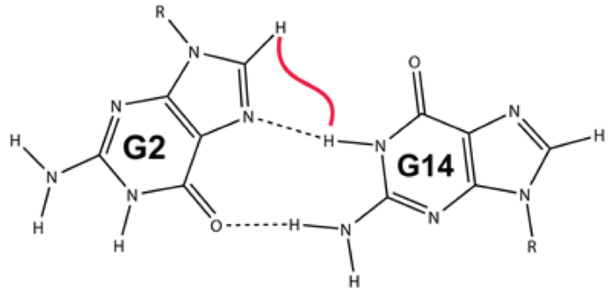

Figure S14. NOESY spectrum of *TP3-T6* (mixing time, 250 ms) at 10°C in 90% H_2O and 10% D_2O, showing interaction between G2 and G14 guanines and G2 with guanines (G3 and G12) forming the top tetrad.

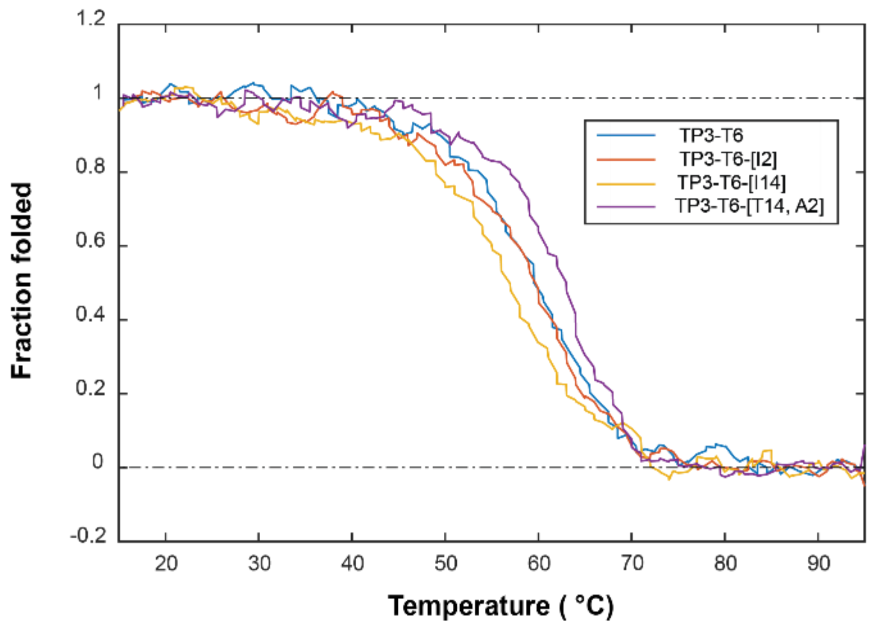

Figure S15. Normalized CD melting curves of *TP3-T6* and sequences mutated at specified positions in 70 mM KCl and 20 mM KPi (pH = 7.0). Cooling curves are displayed with 5-point averaging.
